# Genomic insights into sheath blight resistance: leveraging natural variation in the Bengal-Assam-Aus rice panel for multi-season association mapping

**DOI:** 10.3389/fpls.2026.1890133

**Published:** 2026-09-21

**Authors:** R. Naveenkumar, Siddharth Panda, Challa Vijay Kumar Reddy, S. R. Prabhukarthikeyan, G. Sangeetha, Annamalai Anandan, Licon Kumar Acharya, Jauhar Ali

**Affiliations:** 1 Crop Improvement Division, Indian Council of Agricultural Research (ICAR)-Central Rice Research Institute (CRRI), Cuttack, India; 2 Department of Plant Pathology, Amrita School of Agricultural Sciences, Amrita Vishwa Vidyapeetham, Coimbatore, India; 3 Department of Genetics and Plant Breeding, Institute of Agricultural Sciences (IAS), Siksha O Anusandhan Deemed to be University (DU), Bhubaneswar, India; 4 Department of Genetics and Plant Breeding, College of Agriculture, Odisha University of Agriculture and Technology (OUAT), Bhubaneswar, India; 5 Crop Protection Division, Indian Council of Agricultural Research (ICAR)-Central Rice Research Institute (CRRI), Cuttack, India; 6 Crop Protection Division, ICAR-Indian Institute of Horticulture Research, Bengaluru, India; 7 CAR-National Institute of Seed Science & Technology, Regional Station, Bengaluru, India; 8 ICAR- National Research Institute for Integrated Pest Management, New Delhi, India; 9 Division of Plant Pathology, Indian Council of Agricultural Research (ICAR) - Indian Agricultural Research Institute, New Delhi, India; 10 Rice Breeding Innovation Department, International Rice Research Institute (IRRI), Los Baños, Laguna, Philippines

**Keywords:** AO2, BAAP, BBTI4, DEFL14, G x E, GWAS, protein modeling, sheath blight

## Abstract

**Introduction:**

Sheath blight ranks among rice's most damaging diseases, cutting yield by up to half in severe outbreaks. No source of complete, durable resistance exists, so identifying the genetic basis of partial or quantitative resistance is a priority for breeding.

**Methods:**

We performed GWAS on 130 genotypes from the Bengal-Assam-Aus Panel (BAAP), phenotyped over three seasons viz., Wet 2018, Wet 2019, and Dry 2018-19, scoring disease severity by per cent disease index (PDI) and area under the disease progress curve (AUDPC).

**Results:**

Trait distributions were near-normal with substantial variation across genotypes, pointing to polygenic control of resistance. A strong genotype-by-environment interaction meant combined analysis wasn't appropriate, so we ran GWAS separately per season. This turned up 27 associated loci, three of which on chromosomes 1, 9, and 10 were consistent and high-confidence. The candidate genes underlying these peaks were *LOC_Os01g03360* (BBTI4, a Bowman-Birk protease inhibitor), *LOC_Os09g11790* (DEFL14, a defensin-like peptide), and *LOC_Os10g04860* (AO2, an aldehyde oxidase).

**Discussion:**

The predicted functions include protease inhibition, antifungal activity, and hormone regulation. Instead of relying on a single-gene mechanism, it's advisable to suggest a layered defense response. Collectively, these findings enhance our understanding of the genetic and biochemical aspects of sheath blight tolerance, providing breeders with specific targets to focus on in their efforts.

## 1 Introduction

Rice (*Oryza sativa* L.) is a primary staple food for almost half of the global population, with South and Southeast Asia accounting for approximately 65% of the world’s rice production (Anumalla et al., 2025). With the growing population and rising demand for rice, demand in these regions is projected to increase by up to 87% by 2050 (Radanielson et al., 2018). India ranks second globally in rice production, behind China and contributes approximately 20% of the world’s total rice production (Singh et al., 2019). Globally, rice is grown on around 167.24 million hectares, producing 769.65 million tonnes with an average productivity of 4.6 tonnes per hectare (Panda et al., 2023).

Both biotic and abiotic stresses significantly influence rice productivity. Biotic factors include a range of diseases caused by fungi, bacteria, and viruses. Among them, blast (*Magnaportheoryzae*), brown spot (*Bipolarisoryzae*), and sheath blight (*Rhizoctonia solani*) are considered most damaging (Margani and Widadi, 2018). Sheath blight ranks second after blast as one of the notorious soil-borne diseases of rice (Singh et al., 2019). Yield losses due to this disease can range from 20% to 50%, depending on genotypes, plant growth stage, and prevailing environmental conditions (Dey et al., 2016; Prasad et al., 2020). The infection mechanism of *R. solani* involves a series of steps, such as adhesion to the host surface, penetration, and subsequent colonization. The fungus invades host tissues primarily through stomata and forms specialized structures, such as lobate appressoria or infection cushions, to facilitate entry (Singh and Subramanian, 2017). These infection structures initiate the enzymatic breakdown of the plant cell wall, enabling the pathogen to colonize the tissue and form necrotic lesions. These lesions typically expand to a length of two to three cm and a width of one cm, characterized by a bleached center and purple-brown margins. Over time, lesions may coalesce and spread from leaf sheaths to leaf blades, panicles, and even neighboring tillers (Srinivas et al., 2013). In severe infections, this progression can lead to lodging and plant death; additionally, grain filling is adversely affected due to obstruction of xylem and phloem vessels by fungal structures (Bahuguna et al., 2012). *R. solani* also produces a phytotoxin known as RS toxin, which comprises *N*-acetyl glucosamine, *N-acetyl* galactosamine, mannose, glucose, and various effector molecules, all of which are believed to contribute to the pathogen’s virulence (Zheng et al., 2013).

The development and spread of sheath blight disease are highly influenced by factors such as inoculum load, climatic conditions, and host plant resistance to *R. solani* (Pal et al., 2017). Optimal conditions for disease occurrence include 28°C, 100% relative humidity, and consistently low precipitation (Biswas et al., 2012 and Bhukal et al., 2015). Furthermore, temperature rising to 30°C, along with frequent rainfall and sustained high humidity, significantly enhances disease proliferation (Naveenkumar et al., 2022). *R. solani* persists in the soil primarily as sclerotia or mycelium within infected crop residues. These sclerotia can remain viable in crop debris for up to 3 years, serving as the primary inoculum source for subsequent cropping seasons (Nagaraj et al., 2019). Dissemination of sclerotia between fields occurs mainly through irrigation water, surface runoff, and the movement of contaminated soil (Singh et al., 2019). Under favorable conditions, sclerotia germinate, producing hyphae that spread laterally across plant surfaces, facilitating rapid disease progression (Savary et al., 1995).

The management of *R. solani* has predominantly relied on chemical fungicides; however, extensive use of fungicides raises environmental concerns (Goswami et al., 2019). To align with principles of sustainable agriculture, the identification and utilization of sheath blight-resistant rice genotypes offer a more eco-friendly and economically viable approach to minimizing yield losses and increasing farmers’ income (Dey et al., 2016). Significant efforts have been made to screen rice germplasm across the gene pool for resistance to *R. solani*, resulting in the identification of several promising genotypes. These include Tetep, Teqing, Jasmine 85, Minghui 63, GSOR 310389, GSOR 310475, GSOR 31147, WSS2, Pecos, Zhaiyeqing 8, Jingxi 17, Jarjan, Nepal 8, Nepal 555, BML 27-1, BPL 7-12; BML 21-1, ARC10531, SM801, Gumdhan, Phougak, Wazuhophek, Ngnololasha, RP 2068-18-3-5, Orgoja, and Gopalghosh exhibiting moderate levels of resistance (Yadav et al., 2015; Dey et al., 2016; Parveen and Ali, 2018). Despite these findings, many genotypes identified as moderately resistant have later shown susceptibility in subsequent generations during QTL mapping studies (Dey et al., 2016). This highlights the need for further exploration, as a large portion of global rice germplasm remains untested for sheath blight resistance, offering considerable potential for discovering durable resistance sources.

Extensive QTL mapping efforts have been undertaken to identify genomic regions associated with sheath blight resistance in rice (Li et al., 1995; Zou et al., 2000; Kunihiro et al., 2002; Pinson et al., 2005; Liu et al., 2009; Channamallikarjuna et al., 2010; Liu et al., 2014; Wen et al., 2015; Goad et al., 2020; Bal et al., 2020). Synthesizing results from multiple studies, roughly 100 sheath blight resistance QTLs have been reported across the rice genome (Naveenkumar et al., 2023). Nevertheless, only a limited number of these loci have shown consistent, dependable effects on resistance. To date, only two QTLs, “*qSBR11–1* and *qSb-11^LE^*”, have been fine-mapped (Channamallikarjuna et al., 2010; Zuo et al., 2013). In recent years, gene tagging strategies have shifted from traditional QTL mapping using biparental populations to genome-wide association studies (GWAS) based on linkage disequilibrium (LD) in diverse natural populations. This approach has become a powerful tool for identifying genes of interest and for efficiently utilizing the vast *ex situ* conserved genetic diversity available in global crop germplasm collections (Goldstein and Weale, 2001). To date, only a limited number of studies have used GWAS to identify QTLs associated with resistance to sheath blight in rice. For instance, Chen et al. (2019) conducted a GWAS on 299 rice varieties from the rice diversity panel 1 (RDP1) using 44,000 high-density SNP markers and identified 11 SNPs significantly linked to sheath blight resistance. Similarly, Zhang et al. (2019) performed GWAS on 563 rice accessions using 29, 77,750 SNP markers and detected 132, 562, and 75 SNPs associated with culm length, lesion height, and relative lesion height, respectively, traits correlated with sheath blight resistance. In a more recent study, Fu et al. (2022) identified 23 and 2 SNPs associated with sheath blight resistance using 150 rice cultivars genotyped with 5,173 and 707 SNPs in 2016 and 2017, respectively. These findings highlight the potential of GWAS in uncovering resistance loci across genetically diverse germplasm. Supporting this, Li et al. (2022) analyzed 417 rice accessions from RDP1 using 3.4 million SNPs and identified 21 significant SNPs linked to sheath blight resistance, which corresponded to 18 QTLs. Notably, one of these QTLs, *qShB4*, was novel and had not been reported in previous bi-parental mapping studies. Given the absence of a single gene conferring complete resistance to sheath blight, the current study was undertaken with the specific objectives of identifying new resistance sources through GWAS in the Bengal and Assam Aus Panel (BAAP) developed by Norton et al. (2018). This panel has previously been utilized for diverse stress conditions, such as iron deficiency (Panda et al., 2024), salinity stress (Chen et al., 2020), and hypoxic germination (Vijay Kumar Reddy et al., 2025), root vigor (Anandan et al., 2022) thus presenting as an ideal panel with high-fidelity associations.

## 2 Materials and methods

### 2.1 Planting materials

A total of 130 genotypes from the Bengal and Assam Aus Panel were used in this study, along with two susceptible checks (Pusa Basmati-1 and Tapaswini) and two tolerant checks (Tetep and Jasmine 85). The field evaluation against *R. solani* was conducted at two locations, the Agricultural Research Farm, Institute of Agricultural Sciences, Banaras Hindu University (25°16’N, 82°59’E and 75.5masl), Varanasi, during *Wet season* 2018 and at Central Rice Research Institute, Cuttack (20°27’09’’ N, 85°55’57’’ E, 26 masl) during *Dry season 2018–19* and *Wet season* 2019. The nursery beds were prepared by blending soil, sand, and farmyard manure (FYM) in a 3:1:1 ratio. Seedlings were carefully uprooted and transplanted into the main field after 25–30 days after seeding (DAS), which was laid out in a randomized block design with three replications. Each genotype was planted in a single row 2.0 m long, with 20 cm spacing between plants within the row.

### 2.2 Pathogen inoculation

The strain MTCC12227 (procured from Department of Mycology and Plant Pathology, Institute of Agricultural Sciences, BHU) was used for infection during *Wet season* 2018, while MK478903.1(procured from Crop Protection Division, CRRI, Cuttack, Odisha) was used for screening during *Dry season 2018–19* and *Wet season* 2019. Since the experiments were performed independently at different locations, a direct comparison of virulence between the two isolates was not undertaken. Instead, each isolate had been previously characterized and confirmed as highly virulent under the respective institutional screening program, making it suitable for reliable disease assessment under local experimental conditions. The pathogen was cultured on potato dextrose agar (PDA) at 25 ± 2 °C for 72 to 96 hours. Forty-five-day-old rice plants were inoculated by placing three- to four-day-old sclerotia beneath the leaf sheath, and the infection site was wrapped with moist cotton to maintain humidity to promote fungal development (Singh et al., 2002; Naveenkumar et al., 2022). **Figure 1** illustrates the symptoms developed in the sample plants after inoculation.

**Figure 1:**
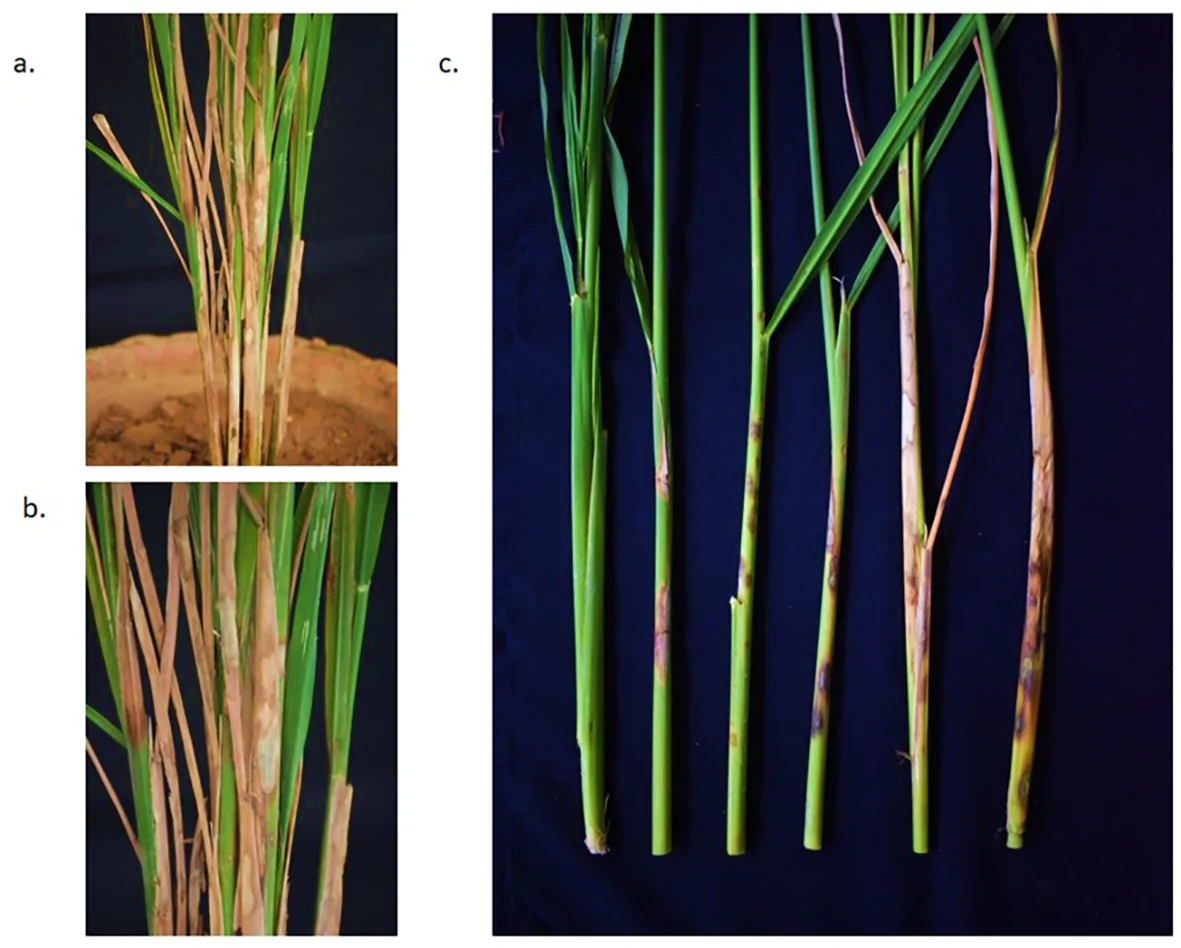
Symptoms of sheath blight caused by *Rhizoctonia solani* in rice following artificial inoculation. **(a, b)** Representative lesions developing on infected rice sheaths and culms after inoculation of 45-day-old plants with *R. solani* sclerotia. **(c)** Representative disease reactions illustrating variation in sheath blight severity among BAAP genotypes, ranging from tolerant to susceptible responses.

### 2.3 Disease assessment

Disease severity for each genotype was assessed weekly at 7,14, 21, and 28 days post-inoculation (dpi) using relative lesion height (RLH) as an indicator. RLH was calculated following the method of Sharma et al. (1990), using the formula,


$$RLH=\frac{Maximum\;height\;at\;which\;lesion\;appear}{Plant\;height\left(cm\right)}\times 100$$

A standard evaluation system (SES) was employed to assign disease severity score on a 0–9 scale, where, 0 = No visible symptom; 1 = lesions confined to the lower 20% of the plant; 3 = lesions covering 20-30% of the plant; 5 = lesions covering 31-45% of the plant; 7 = lesions covering 46-65% of the plant and 9 = more than 65% of the plant height affected (International Rice Research Institute (IRRI), 2014). The per cent disease index (PDI) was calculated using the formula proposed by Wheeler (1969)


$$PDI=\frac{Sum\;of\;all\;rating}{Total\;no.\;of\;observations\times Maxium\;rating\;scale}\times 100$$

To quantify disease progression over time, the area under the disease progression curve (AUDPC) was computed from disease severity percentages recorded at weekly intervals. The formula imposed by Shaner and Finney (1977), was used,


$$AUDPC=\sum_{i=1}^{n}\left\{\left[\left(Xi+1\;+\;Xi\right)\right]/2\;x\;\left(ti+1\;-\;ti\right)\right\}$$

where;

X_i_ = disease index at the i^th^ observation
t_i_= time in days post inoculation (dpi) at the i_th_ observation and
n = total number of observations

### 2.4 Statistical analysis

Descriptive statistics, ANOVA and Pearson’s correlation analyses were calculated for Plant height, Days to 50% flowering, Tiller number, Panicle length, Mean PDI, and AUDPC across all three seasons using GRAPES (Gopinath et al., 2020). The graphs and plots were developed using Minitab v 19.1 and the SRPlot online platform (Tang et al., 2023).

### 2.5 Genome-wide association mapping

Genome-wide association (GWA) analysis was carried out on the BAAP panel using the PIQUE pipeline, which preprocesses genotype and phenotype datasets before parallel implementation of the EMMAX mixed-model association analysis (Norton et al., 2018). GWA mapping was performed using phenotypic data and the publicly available BAAP genotype dataset comprising 2,053,863 high-quality SNPs previously generated and processed by Norton et al. (2018). Single-nucleotide polymorphisms (SNPs) with a minor allele frequency (MAF) below 0.05 were excluded, and markers with more than 5% missing genotype calls were removed prior to association analysis. These quality-control filters were applied to retain informative polymorphic markers while minimizing spurious associations arising from rare alleles and excessive missing genotype data. GWA mapping was performed using phenotypic data and SNPs with EMMAX method to account for both population structure and kinship, with a significance cutoff of p< 0.0001, following Norton et al. (2018). To assess the association between each SNP and phenotype across all cultivars, a mixed-effects model was employed. The model accounted for both population structure and cryptic kinship: population structure was incorporated as fixed-effect covariates using the first five principal components derived from a PCA of all 2,053,863 BAAP SNPs across cultivars, computed via EIGENSTRAT smartpca (Price et al., 2006), while genetic similarity between individuals was captured as a random effect through a kinship matrix quantifying the proportion of shared genotypes across all SNPs for each pair of cultivars (IBS values). Together, these fixed and random effects were integrated into GWAS models across all cultivars to ensure robust control for confounding structure and relatedness. The false discovery rate (FDR) was estimated using the Benjamini–Hochberg adjusted p-values (Benjamini and Hochberg, 1995), and SNP associations were considered putative when they passed a 10% FDR threshold (McCouch et al., 2016).

Following the GWA analysis, clump analysis was performed to group multiple significant SNPs (p< 0.0001) representing a single QTL using the PLINK software (Purcell et al., 2007) with the parameters “‐‐clump-p1 0.0001 –clump-p2 0.0001 –clump-r2 0.3 –clump-kb 243.” Singleton SNPs with p< 0.0001 were excluded if no additional SNPs within the linkage disequilibrium (LD) decay window met the same significance threshold. Clump analysis was conducted independently for each trait, and clumps identified across all traits were then compared by genomic position. Clumps detected at the same locus or within a 243 kb window—the estimated genome-wide LD decay in the BAAP panel (Norton et al., 2018)—were merged. Subsequently, FDR-adjusted significance values were examined for all SNPs within QTL regions, and only those QTLs containing at least one significant SNP (p< 0.0001) that also met the 10% FDR threshold were retained.

### 2.6 Identification of candidate genes and protein structure assessment

For each QTL, a genomic interval of 243 kb surrounding the peak SNP was delineated, and gene annotations within these regions were retrieved from the Rice Genome Annotation Project (RGAP, release 7; http://rice.plantbiology.msu.edu/). Genes annotated as “(retro)transposon,” “hypothetical,” or “unknown” were excluded from further consideration. Candidate genes were prioritized based on (i) their physical position within the LD interval, (ii) functional annotation and Gene Ontology classification in RGAP, (iii) previous reports implicating the gene or its homologues in disease resistance or biotic stress responses, and (iv) the presence of non-synonymous SNPs defining distinct haplotypes associated with significant differences in sheath blight response. Among the prioritized candidates, three genes LOC_Os01g03360 (BBTI4), LOC_Os09g11790 (DEFL14), and LOC_Os10g04860 (AO2) were selected for protein structural analysis because they represented the strongest and most biologically plausible candidate genes from the three highest-confidence GWAS loci identified across the independent seasonal analyses. The identified genes’ coding sequences were extracted from the RGAP database and processed to study the nature of their proteins, composition, and stability using the Protparam tool on the Expasy server (Gasteiger et al., 2005) and the Rice Seed Nuclear Protein Database. The protein modeling and its structure assessment using the Ramachandran Plot were accomplished using the SWISS-MODEL repository (Waterhouse et al., 2024). Protein structural analyses were performed as complementary evidence to assess whether the encoded proteins possessed structural and physicochemical characteristics consistent with their predicted biological functions. These analyses were intended to support the biological plausibility of the prioritized candidate genes rather than to provide functional validation of the GWAS associations.

## 3 Results

### 3.1 Phenotyping the panel and population structure

The panel population’s distribution showed a broad range of variation across three growing seasons (*Wet season* 2018, *Wet season* 2019, and *Dry season 2018-19*) (**Supplementary Table 1**). The mean and median values were generally consistent across most traits, except for AUDPC and mean PDI, which showed noticeable differences across seasons (**Supplementary Table 2**). These variations suggested that the population followed a normal distribution frequency. Among the evaluated traits, plant height exhibited strong negative skewness (<-1) during *Wet season* 2018 and *Dry season 2018-19*, while panicle length also showed negative skewness during *Dry season 2018-19*. In contrast, the remaining traits showed positive skewness. Kurtosis values for most traits fell within the range of -1 to +1, with exceptions such as AUDPC (6.01), days to 50% flowering (4.87) in *Wet season* 2018. Similarly, in *the Dry season 2018-19*, higher kurtosis values were recorded for days to 50% flowering (5.64), plant height (1.36), and tiller number (2.05). These results indicate that the population frequency distribution was predominantly leptokurtic. The coefficient of variation (CV) further supported this observation, with values ranging from 0.07% (days to 50% flowering) to 0.35% (AUDPC) during *the 2018 wet season*. In the *Dry season 2018-19*, CV values varied from 0.07% (days to 50% flowering) to 0.28% (tiller number per plant), while in the *Wet season* 2019, they ranged between 0.06% (days to 50% flowering) and 0.32% (tiller number per plant).

The pooled analysis of variance was performed for the six traits for the three seasons evaluated against sheath blight (**Supplementary Table 3**). The pooled analysis of variance (ANOVA) revealed highly significant genetic variability among the 130 genotypes (p< 0.001) for all evaluated traits, including disease resistance (AUDPC and Mean PDI) and agro-morphological characters (flowering, panicle length, plant height, and tiller number). This strong genetic signal confirms the suitability of the population for genome-wide association mapping. Furthermore, the Genotype x Location (GxL) interaction was highly significant (p< 0.001) across all traits, indicating that the performance of the genotypes, particularly their resistance to sheath blight, was significantly influenced by the environmental conditions of the three test locations.

The Pearson correlation analysis was conducted to assess relationships among traits associated with sheath blight resistance in rice across seasons (**Supplementary Table 4**). In *Wet season* 2018, AUDPC showed a strong positive association with days to heading (0.307***) and mean PDI (0.986***), but a significant negative association with plant height (-0.400***). Similarly, mean PDI exhibited positive correlations with days to heading (0.283***) and negative correlations with plant height (-0.449***). Panicle length was positively correlated with both plant height (0.337***) and days to heading (0.231**). During *Dry season 2018-19*, AUDPC again had a highly significant positive correlation with mean PDI (0.977***) and a negative correlation with plant height (-0.237***). Panicle length showed strong associations with plant height (0.469***) and days to heading (0.189*). In *Wet season* 2019, AUDPC was positively correlated with mean PDI (0.993***) and negatively correlated with plant height (-0.182**). Meanwhile, panicle length maintained a strong positive relationship with plant height (0.630***), while a negative association with days to heading (-0.177*).

The population structure of the BAAP has been previously characterized using genome-wide SNP markers, with five distinct population subgroups (K = 5) identified using fastSTRUCTURE and ADMIXTURE, consistent with earlier STRUCTURE analysis (Norton et al., 2018). This previously reported population structure was considered in the present GWAS analysis, and the first five principal components derived from the 2,053,863 BAAP SNPs were incorporated as fixed-effect covariates in the EMMAX model, while an Identity by State (IBS)-based kinship matrix was included as a random effect to account for genetic relatedness among the genotypes.

### 3.2 Genome-wide association studies

The GWA mapping of the BAAP lines for sheath blight tolerance, as measured by AUDPC, highlighted significant genomic segments across different chromosomes. This process was done for all three seasons evaluated in this study. The clump analysis of the significant peak SNPs identified a total of 27 genomic loci distributed across all chromosomes except chromosome 12, in the three different seasons (**Supplementary Table 5**). The Manhattan plots for these associations are presented in **Figure 2**. Of these, the one highest peak from each of the three seasons was further considered for studying the region in LD. The three peaks chosen for further study were at 1308097 bp on chromosome 1 (*Wet season*, 2018), 2230658 bp on chromosome 10 (*Wet season*, 2019), and 6626712 bp on chromosome 9 (*Dry season*, 2018-19) (**Table 1**). The following sections further dissect the LD region in association with the above-mentioned peaks.

**Figure 2:**
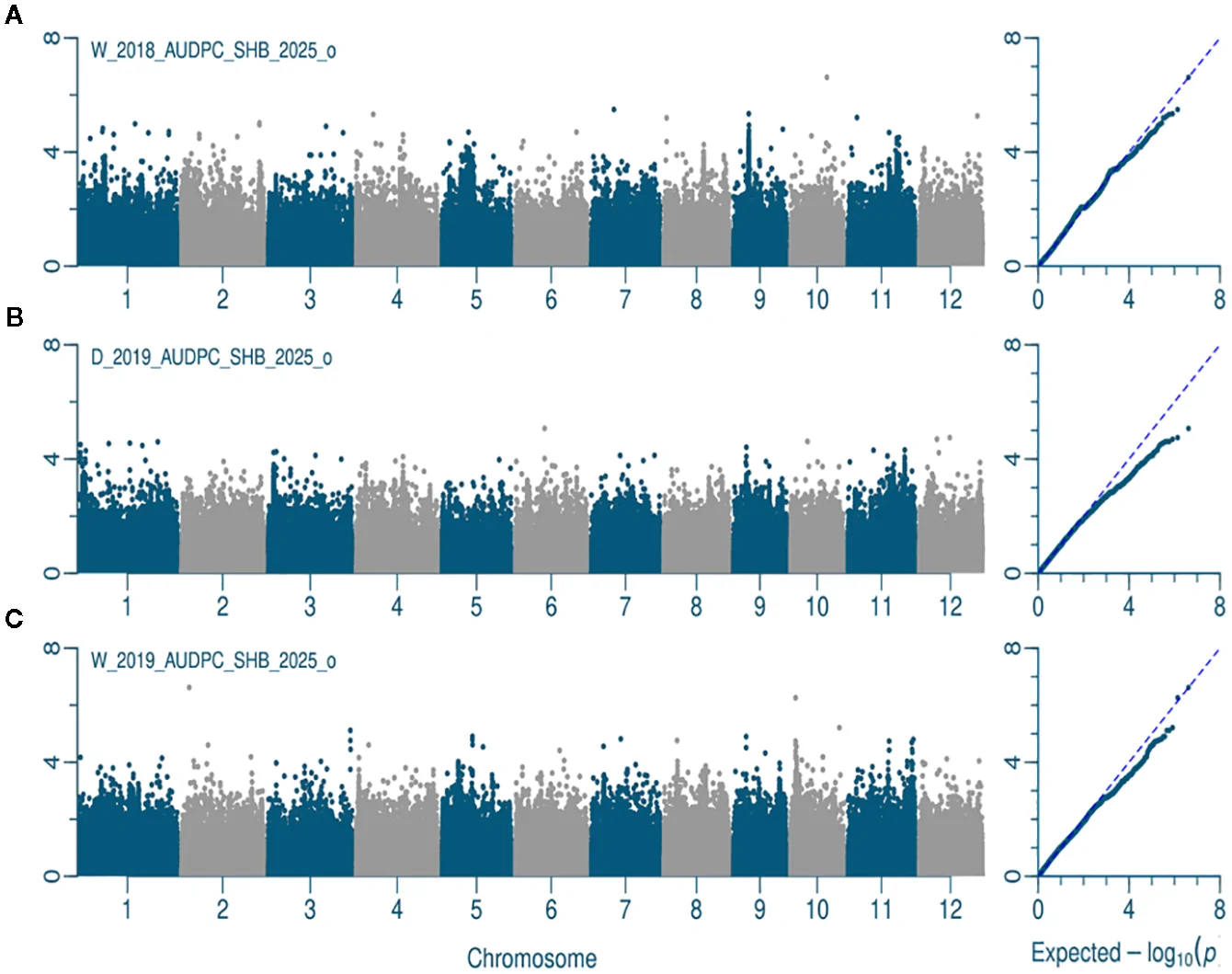
Genome-wide association mapping of area under the disease progress curve (AUDPC) for sheath blight resistance in the Bengal–Assam Aus Panel (BAAP). Manhattan plots show genome-wide SNP associations for **(A)** Wet season 2018, **(B)** Dry season 2018–19, and **(C)** Wet season 2019 using approximately 2.05 million SNPs. Each point represents a SNP plotted according to chromosomal position and –log10(P). The horizontal line indicates the genome-wide significance threshold (P< 0.0001). Corresponding quantile–quantile (QQ) plots compare observed and expected P-value distributions; deviation from the diagonal at the upper tail indicates true marker–trait associations after controlling for population structure and kinship.

**Table 1:** List of putative candidate genes identified for AUDPC against sheath blight in rice across three different seasons.

Season	Chr	Peak SNP (in bp)	LD (in bp)	Putative candidate	Gene stretch	Strand
*Wet season*, 2018	9	6626712	6383712 - 6869712	LOC_Os09g11790.1 (DEFENSIN-LIKE 14)	6594470 - 6594871	+
*Wet season*, 2019	10	2230658	1987658 – 2473658	LOC_Os10g04860.1 (ALDEHYDE OXIDASE 2)	2358790 - 2358790	+
*Dry season*, 2018-19	1	1308097	1065097 – 1551097	LOC_Os01g03360.1 (BOWMAN-BIRK INHIBITOR 3-3)	1354802 - 1353629	–

### 3.3 AUDPC for sheath blight, *Wet season* 2018

The most significant peak associated with AUDPC under sheath blight infection in the *Wet season* 2018 was at 6626712 bp on chromosome 9. This region in LD with the peak (6383712–6869712 bp) contained 61 genes, excluding hypothetical proteins, transposons, and retrotransposons (**Supplementary Table 6**). The gene LOC_Os09g11790.1 was chosen as the putative candidate gene. This gene is annotated as Defensin-Like 14, harboring four annotated Defensin and Defensin-like DEFL family (OsDEFL14) non-synonymous SNPs at 6594512, 6594536, 6594710, and 6594788 bp, with G/T, C/T, G/T. and C/T nucleotide polymorphisms respectively. These polymorphisms segregated the panel into three groups: Hap A, with an AUDPC mean of 457.4, had the highest AUDPC values, followed by Hap B and Hap C, with AUDPC values of 352.3 and 314.9, respectively. Haplotype comparisons revealed that Hap B and Hap C both showed significant reductions relative to Hap A, with mean differences of -105.1 (SE = 40.6, 95% CI, -209.7 to -0.6, p = 0.048) and -142.5 (SE = 44.8, 95% CI, -255.0 to -29.9, p = 0.011), respectively (**Figure 3**). In contrast, the difference between Hap C and Hap B (-37.3, SE = 22.8, 95% CI, -95.6 to 20.9, p = 0.257) was not significant, indicating that both Hap B and Hap C differ from Hap A but not from each other.

**Figure 3:**
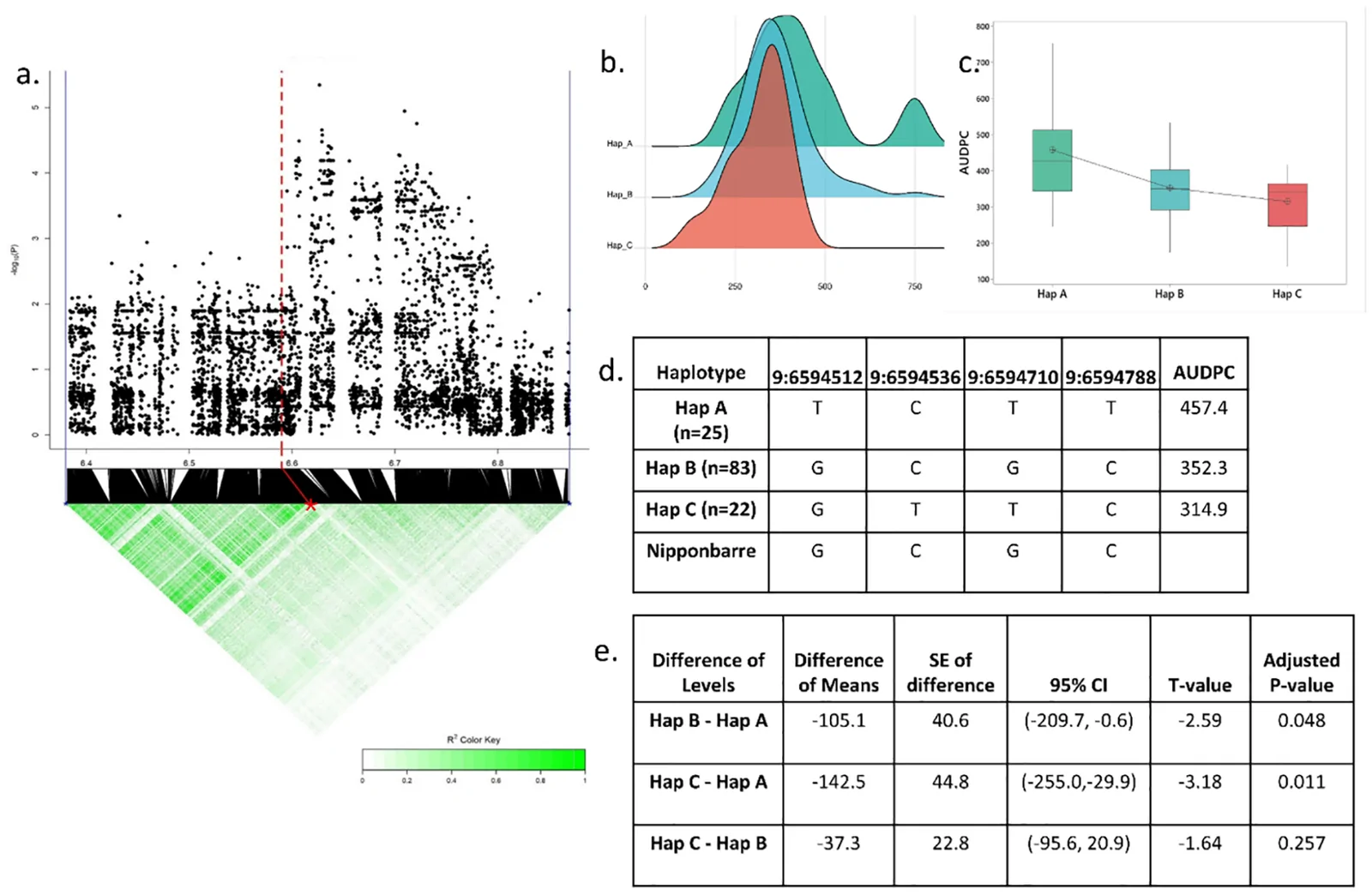
Fine mapping and haplotype analysis of the chromosome 9 linked to AUDPC for sheath blight resistance locus containing LOC_Os09g11790 (DEFL14). **(a)** Local Manhattan plot and linkage disequilibrium (LD) heat map surrounding the peak SNP at 6,626,712 bp. The blue line indicates the 243 kb LD interval surrounding the lead SNP, while the red dashed line marks the position of the candidate gene LOC_Os09g11790 (DEFL14). **(b)** Distribution of AUDPC values among the three DEFL14 haplotypes. **(c)** Boxplots comparing AUDPC across haplotypes. **(d)** Non-synonymous SNPs defining the three DEFL14 haplotypes. **(e)** Pairwise Games–Howell comparisons of haplotype means. Asterisks indicate statistical significance (P< 0.05, P< 0.01, P< 0.001, P< 0.0001; ns = not significant). Red asterisks indicate the positions of the genes.

### 3.4 AUDPC for sheath blight, *Wet season* 2019

The peak SNP with the highest significance associated with this season was at 2230658 bp on chromosome 10, with LD extending from 1987658 bp to 2473658 bp. A closer look at this region revealed 54 different genes after filtering out hypothetical proteins, transposons, and retrotransposons (**Supplementary Table 7**). Of these, LOC_Os10g04860.1 was chosen as the putative candidate gene influencing AUDPC. The gene is annotated as Aldehyde Oxidase 2 (OsAO2) and harbored five nsSNPs; 2362651 (T/C), 2363290(A/C), 2364066(G/A), 2365632(G/A), and 2366330bp (A/G). These nsSNPs grouped the panel into three haplotypes. Hap B (852.8) had the highest AUDPC value, followed by Hap C (715.6) and Hap A (642.3) (**Figure 4**). Haplotype comparisons showed that both Hap B and Hap C had significantly higher trait values compared to Hap A, with mean differences of 210.5 (SE = 69.0, 95% CI, 21.2–399.8, p = 0.030) and 73.3 (SE = 27.0, 95% CI, 3.1–143.4, p = 0.040), respectively (**Figure 4**). The difference between Hap C and Hap B (-137.3, SE = 68.6, 95% CI, -324.9–50.4, p = 0.162) was not significant, indicating that while both Hap B and Hap C are elevated relative to Hap A, they do not differ significantly from each other.

**Figure 4:**
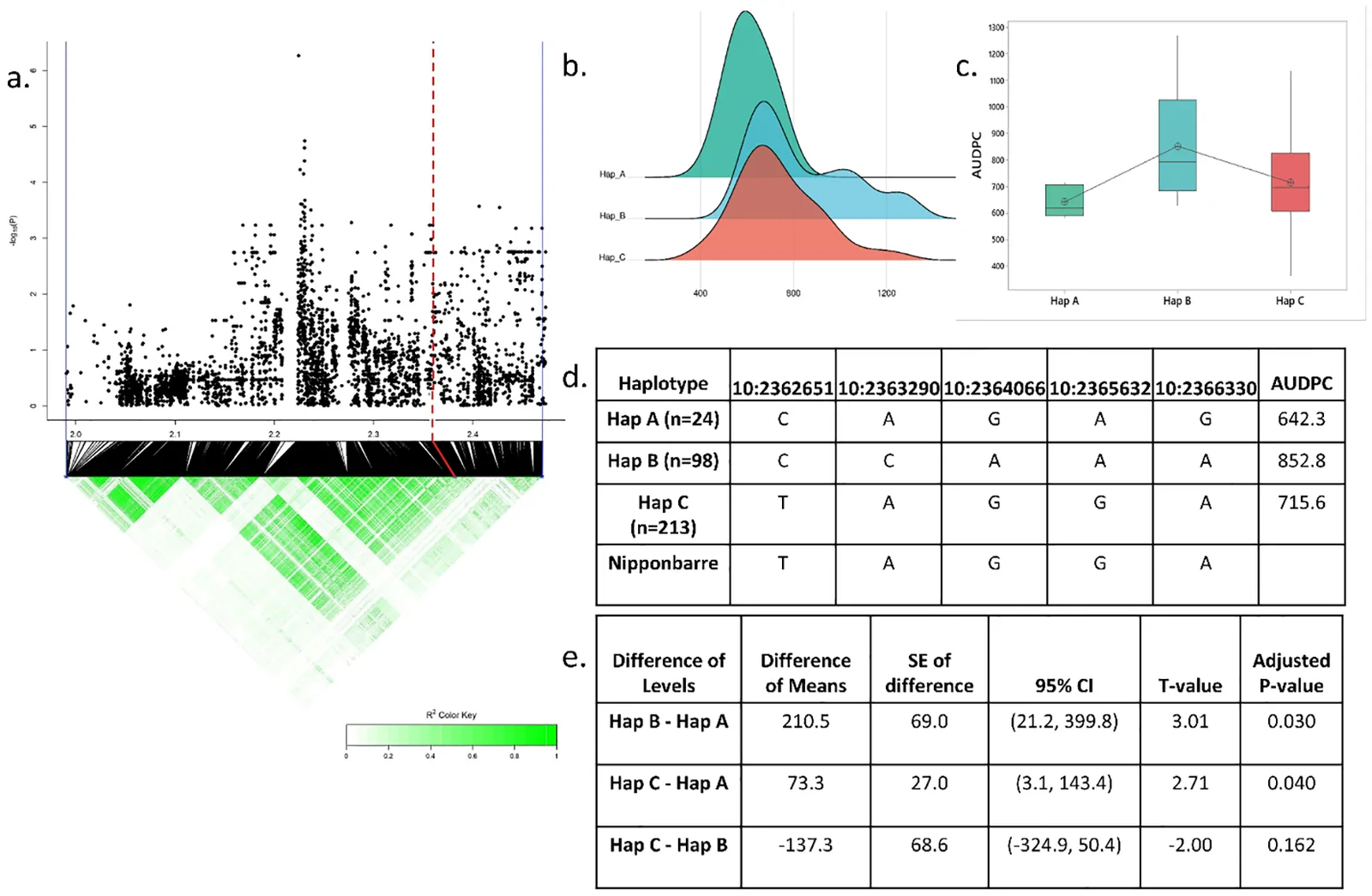
Fine mapping and haplotype analysis of the chromosome 10 linked to AUDPC for sheath blight resistance locus containing LOC_Os10g04860 (OsAO2). **(a)** Local Manhattan plot and linkage disequilibrium (LD) heat map surrounding the peak SNP at 2,230,658bp. The blue line indicates the 243 kb LD interval surrounding the lead SNP, while the red dashed line marks the position of the candidate gene LOC_Os10g04860 (OsAO2). **(b)** Distribution of AUDPC values among the three OsAO2 haplotypes. **(c)** Boxplots comparing AUDPC across haplotypes. **(d)** Non-synonymous SNPs defining the three OsAO2 haplotypes. **(e)** Pairwise Games–Howell comparisons of haplotype means. Asterisks indicate statistical significance (P< 0.05, P< 0.01, P< 0.001, P< 0.0001; ns = not significant). Red asterisks indicate the positions of the genes.

### 3.5 AUDPC for sheath blight, *Dry season 2018-19*

The highest peak SNP associated with AUDPC for sheath blight in *Dry season* 2018 was on chromosome 1 at 1308097bp. The associated region of LD stretched from 1065097–1551097 bp harboring 64 genes excluding hypothetical proteins, transposons, and retrotransposons (**Supplementary Table 8**). The gene LOC_Os01g03360.1, annotated as BOWMAN-BIRK INHIBITOR 3-3 (OsBBPI). The gene had four nsSNPs; 1354397, 1354507, 1354574, and 1354617 with A/G, C/G, C/G, and C/G polymorphisms, respectively. The entire panel was divided into two haplotypes: HapB (365.8) and HapA (426.4).HapA showed a significant increase in AUDPC values compared to HapB, with a mean difference of 60.6 (SE=11.3;95%CI,36.5–84.8; p=0.008),indicating a strong, statistically meaningful effect of this haplotype (**Figure 5**). The superior haplotype: HapB was significantly different from HapA, which had a similar allelic make up as that of the wild type Nipponbarre genome.

**Figure 5:**
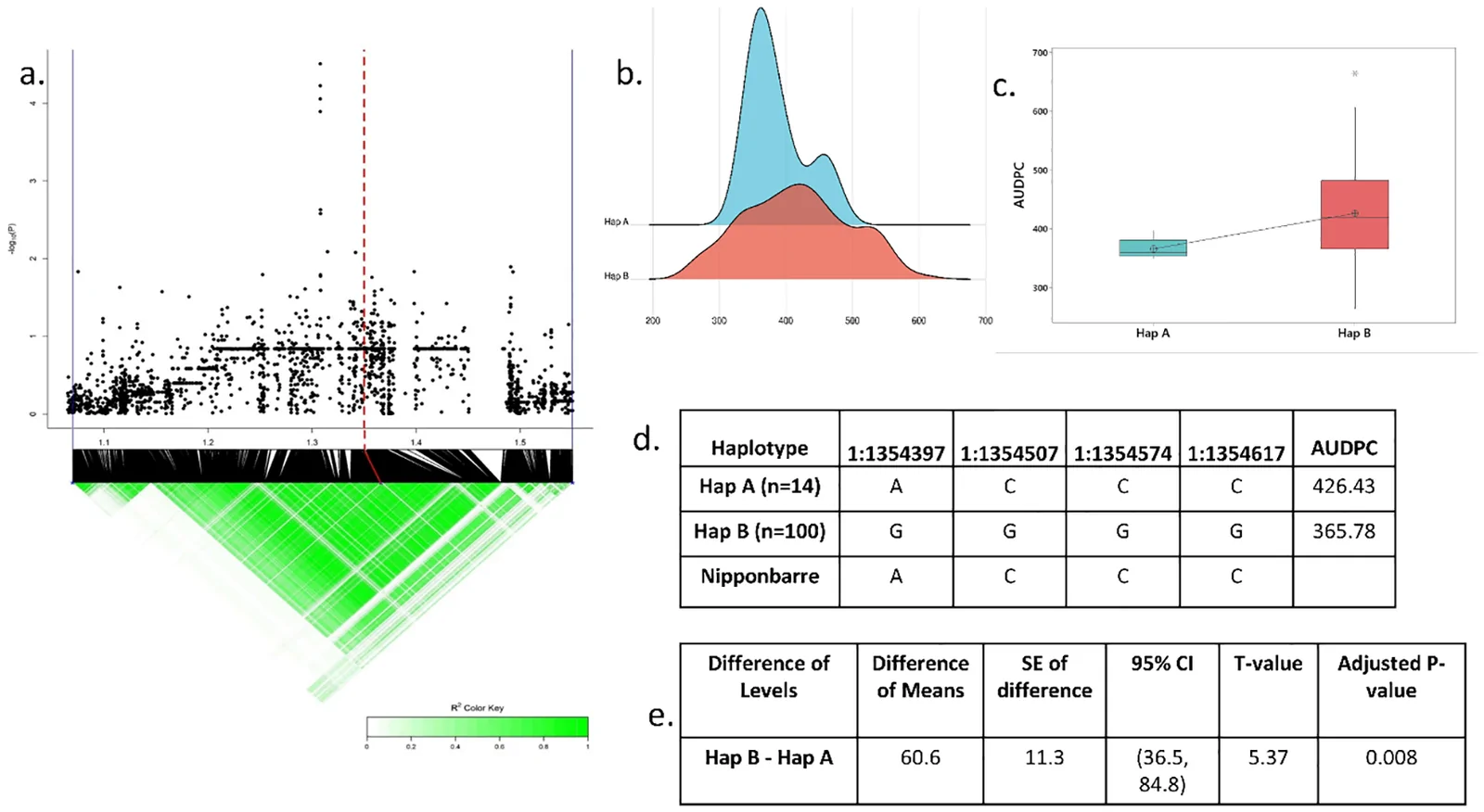
Fine mapping and haplotype analysis of the chromosome 1 linked to AUDPC for sheath blight resistance locus containing LOC_Os01g03360 (OsBBPI). **(a)** Local Manhattan plot and linkage disequilibrium (LD) heat map surrounding the peak SNP at 1308097bp. The blue line indicates the 243 kb LD interval surrounding the lead SNP, while the red dashed line marks the position of the candidate gene LOC_Os01g03360 (OsBBPI). **(b)** Distribution of AUDPC values among the three OsBBPI haplotypes. **(c)** Boxplots comparing AUDPC across haplotypes. **(d)** Non-synonymous SNPs defining the two OsBBPI haplotypes. **(e)** Pairwise Games–Howell comparisons of haplotype means. Asterisks indicate statistical significance (P< 0.05, P< 0.01, P< 0.001, P< 0.0001; ns = not significant). Red asterisks indicate the positions of the genes.

## 4 Discussion

The evaluation of the Bengal-Assam-Aus Panel against sheath blight infection revealed substantial phenotypic variation across the three growing seasons, indicating a broad genetic base and diverse responses to disease pressure. The near-normal distribution of most traits, as supported by skewness and kurtosis values, suggested polygenic control of sheath blight resistance and related agronomic traits. The high kurtosis observed for AUDPC and days to 50% flowering in *Wet season* 2018, and for plant height and tiller number in *Dry season 2018-19*, reflected the presence of a few extreme genotypes with distinct responses to sheath blight stress. The moderate coefficients of variation across traits further confirmed the stability and reliability of the dataset for downstream analyses such as GWAS. The pooled ANOVA summarized the performance of evaluated rice lines across three environments, *Wet season* 2018, *Wet season* 2019, and *Dry season 2018-19*. The analysis revealed significant variation among genotypes and environments for all studied traits, indicating the presence of both genetic diversity and environmental influence. The combined ANOVA further showed that genotype × environment interactions were substantial for several traits, suggesting differential responses of lines across seasons. Running separate GWAS per season was the appropriate way to detect environment-specific QTLs or alleles, which might be crucial for adaptation to specific conditions. Correlation analysis provided key insights into the relationships among traits. The strong positive association between AUDPC and mean PDI across all seasons confirmed their interdependence as reliable indicators of sheath blight severity, while the consistent negative correlation of both traits with plant height suggested that taller genotypes exhibited reduced susceptibility to sheath blight. The positive associations among panicle length, plant height, and days to heading indicated coordinated growth patterns that influence morphological resistance. Overall, the results highlight a complex but consistent pattern of relationships, implying that sheath blight resistance in the Bengal-Assam-Aus panel is governed by multiple interacting factors influenced by both plant architecture and phenological traits.

### 4.1 LOC_Os09g11790 (DEFL14) – a defensin/defensin-like peptide for sheath blight tolerance in rice

LOC_Os09g11790 (DEFL14) encodes a defensin/defensin-like family protein located on chromosome 9 (Silverstein et al., 2007; Carvalho and Gomes, 2009). Plant defensins are small, cysteine-rich peptides with broad antifungal activity and roles in innate immunity (Thomma et al., 2002). They belong to the RGAP defensin/DEFL family and are reported pathogenesis-related (PR) proteins induced by biotic and abiotic stresses (Silverstein et al., 2007; Carvalho and Gomes, 2009). These proteins can induce direct antifungal effects while also participating in signaling at pathogen invasion sites, consistent with quantitative tolerance mechanisms against *Rhizoctonia solani*. *Rhizoctonia solani* infection involves fungal colonization and lesion expansion at the sheath interface. DEFL14 may act directly as an antifungal peptide, disrupting fungal membranes, or indirectly by modulating reactive oxygen species (ROS) and defense signaling pathways, including jasmonate (JA) and ethylene (ET)-mediated cascades (Thomma et al., 2002). DEFL14 is a biochemically and mechanistically credible candidate for rice defense against sheath blight (Silverstein et al., 2007). Its small, disulfide-stabilized, extracellular nature allows direct antifungal action and thereby, potential modulation of ROS or defense signaling at the infection site (Thomma et al., 2002). Integrating DEFL14 into multi-gene resistance frameworks could enhance sheath blight tolerance by providing both a biochemical barrier and a signaling component of innate immunity (Carvalho and Gomes, 2009).

The gene’s coding sequence was extracted, and the corresponding protein was modeled and further studied for its stability and physiochemical properties using the SWISS Model, Protparam and Rice Seed Nuclear Protein Database. The analyses revealed that the gene encodes a protein of ~8.3 kDa cysteine-rich peptide (**Figure 6**) with high sulfur content (11 cysteines), enabling multiple disulfide bonds that stabilize its tertiary structure and confer resistance to proteolysis (Creighton, 1993; Marino and Gladyshev, 2010). The peptide is mildly hydrophilic (GRAVY = -0.195), with a nearly neutral charge profile (8 acidic vs. 9 basic residues) supporting stable folding and limited nonspecific electrostatic interactions, typical of short signaling or defense peptides with apoplastic localization, with an NLS of -0.47 (Rawlings et al., 2018). Structural quality assessment yielded a QMEANDisCo score of 0.42 ± 0.10 and a QMEAN Z-score of −2.19, indicating an acceptable predicted model suitable for qualitative structural interpretation and comparative analysis. The structure exhibits reasonable stereochemical quality, with a MolProbity score of 2.34, suggesting satisfactory overall refinement (Chen et al., 2010). A clash score of 4.10 indicates few steric overlaps, reflecting good atomic packing. The Ramachandran Favored value of 85.90% shows that most residues adopt energetically stable conformations, though some local deviations remain that could benefit from minor optimization (Sobolev et al., 2020).

**Figure 6:**
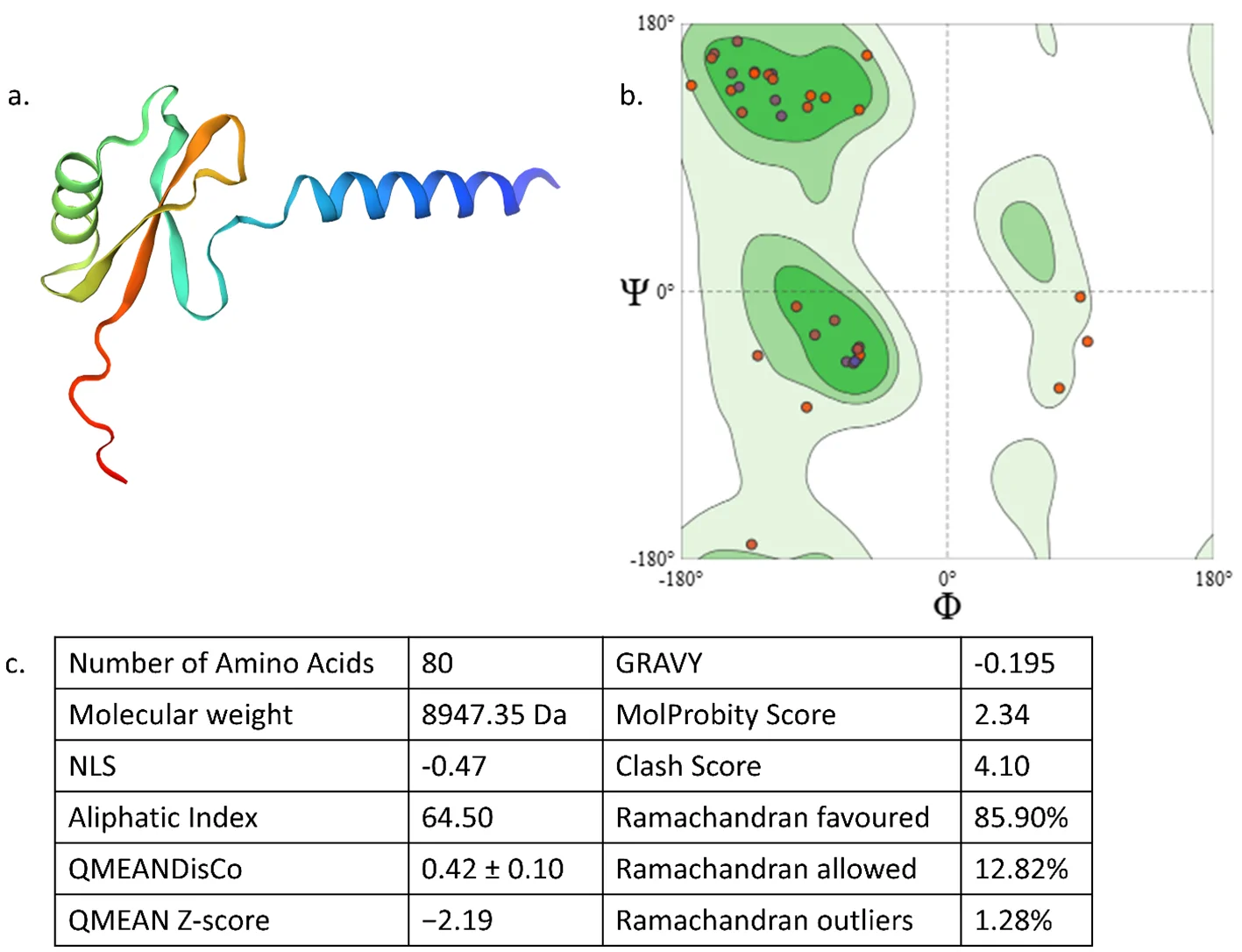
*In silico structural characterization of the protein encoded by the gene* LOC_Os09g11790 (DEFL14). **(a)** Predicted 3D structure of the 80-amino-acid peptide (~8.9 kDa) generated using SWISS MODEL, showing a compact, disulfide-stabilized fold and an extended α-helical region at the C-terminus **(b)** Ramachandran plot illustrating the stereochemical quality of the modeled structure **(c)** Summary of key physicochemical and validation parameters.

### 4.2 LOC_Os10g04860 – an aldehyde oxidase implicated in hormone crosstalk and sheath blight tolerance in rice

LOC_Os10g04860 encodes a putative aldehyde oxidase (OsAO2) located on chromosome 10 (Shi et al., 2021; Colasuonno et al., 2017). Aldehyde oxidases are molybdoenzymes that catalyze the oxidation of aldehydes to carboxylic acids and are critical in the final step of abscisic acid (ABA) biosynthesis, converting abscisic aldehyde to ABA (Shi et al., 2021; Colasuonno et al., 2017; Seo et al., 2000). These enzymes also interface with auxin metabolism, linking ABA and auxin signaling pathways that are central to stress and disease response in the plants (Colasuonno et al., 2017; Wu et al., 2025; Shi et al., 2021). In pathosystems involving necrotrophic pathogens like *Rhizoctonia solani*, elevated ABA can antagonize jasmonic acid (JA) and ethylene (ET)-mediated defense signaling, increasing susceptibility, while reduced ABA levels enhance JA/ET-dependent defenses, limiting lesion development (Verma et al., 2016; Li et al., 2021). Thus, LOC_Os10g04860 likely functions as a hormonal rheostat, influencing the outcome of the host - pathogen interactions (Shi et al., 2021; Yang et al., 2024). It represents a hormonally integrative candidate for sheath blight tolerance. Functional manipulation of this AO/AAO gene could fine-tune hormonal homeostasis, providing leverage for engineering broad-spectrum, hormone-mediated resistance in rice (Shi et al., 2021; Colasuonno et al., 2017; Li et al., 2021).

This gene was further processed for protein studies and revealed a large, cytosolic molybdoenzyme, which exhibits high molecular stability (**Figure 7**). The predicted protein model exhibited a QMEANDisCo global score of 0.69 ± 0.05, indicating moderate agreement between the model and experimentally determined protein structures of similar size. The QMEAN Z-score of −3.63 suggests that while the overall fold is plausible, certain regions of the model may possess lower structural reliability and should be interpreted with caution. The protein has an aliphatic index of 83.44, indicating considerable thermostability (Ikai, 1980), and a GRAVY value of -0.195, reflecting nearly neutral hydropathicity typical of soluble cytosolic enzymes. Its charge distribution is slightly acidic, with 142 acidic residues versus 127 basic residues, supporting cytoplasmic solubility and cofactor interactions (Hille, 2002). The structure demonstrates excellent stereochemical quality, with a MolProbity score of 1.85 indicative of high-resolution refinement (Chen et al., 2010). The very low clash score (3.90) confirms minimal steric conflicts between atoms. Additionally, 91.26% of residues falling in Ramachandran-Favoured regions reflects well-optimized backbone geometry and overall structural reliability (Sobolev et al., 2020). These features describe a stable, cofactor-binding oxidase capable of functioning efficiently under stress conditions that elevate ABA synthesis (Schwarz and Mendel, 2006).

**Figure 7:**
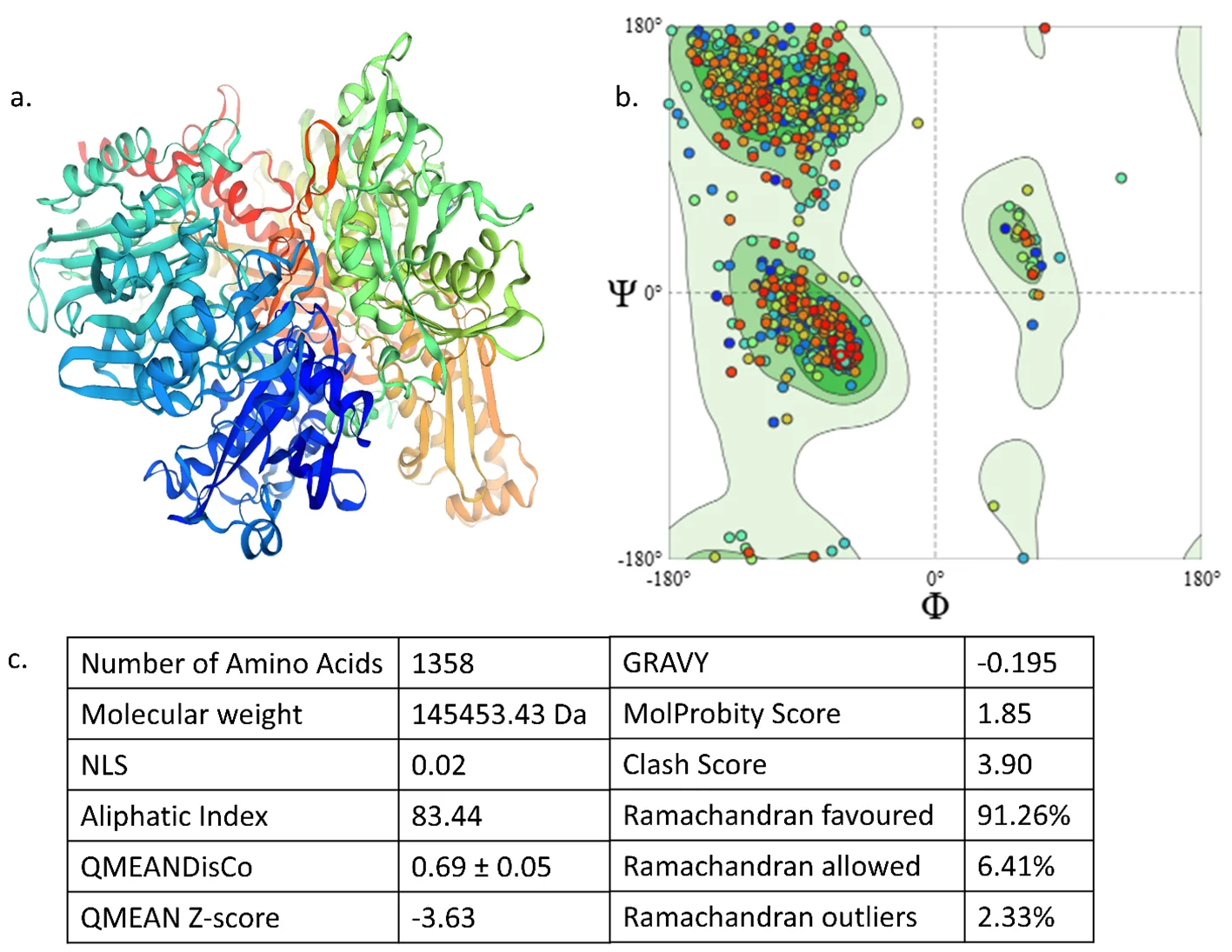
*In silico structural characterization of the protein encoded by the gene* LOC_Os10g04860 OsAO2). **(a)** Predicted 3D structure of the 1358-amino-acid peptide (~145.4kDa) generated using SWISS MODEL, revealing a multi-domain organization **(b)** Ramachandran plot illustrating the stereochemical quality of the modeled structure **(c)** Summary of key physicochemical and validation parameters.

### 4.3 LOC_Os01g03360 - a Bowman–Birk protease inhibitor mediating protease-dependent defense against sheath blight in rice

LOC_Os01g03360 (BBTI4) encodes a member of the Bowman–Birk type protease inhibitor (BBI) family, located on chromosome 1 of Oryza sativa (Qu et al., 2003; Gitlin-Domagalska et al., 2020; Rakwal et al., 2001). Bowman–Birk inhibitors are small, cysteine-rich proteins that act as defense effectors, capable of inhibiting serine proteases secreted by pathogens (Gitlin-Domagalska et al., 2020). By neutralizing pathogen-derived proteases, they restrict tissue colonization and delay the spread of infection (Zhang et al., 2020; Lone et al., 2021). Pathogens such as *Rhizoctonia solani* deploy proteolytic enzymes to degrade host cell walls and suppress defense signaling (Malefo et al., 2020). BBTI4 may counteract this by binding to pathogen proteases, thereby limiting tissue maceration (Zhang et al., 2020),. The gene belongs to a multigene cluster of BBI-type trypsin and chymotrypsin inhibitors, a hallmark of stress-adaptive protein families implicated in nonhost resistance and quantitative defense (Qu et al., 2003; Gitlin-Domagalska et al., 2020). It represents a biochemically viable candidate gene for protease-mediated defense in rice. Its cysteine-rich, disulfide-stabilized structure supports resilience in the oxidative apoplast, while its biochemical flexibility enables quick deployment during infection (Malefo et al., 2020). BBTI4 likely operates as both a direct biochemical barrier against pathogen proteases and a regulatory element in hormone-mediated defense signaling (Nahar et al., 2011; Xie et al., 2022). In quantitative resistance contexts, such inhibitors may fine-tune ROS homeostasis, limit lesion expansion, and maintain cell integrity around infection sites.

The protein model generated from the SWISS Model web tool the omics exploration from the ProtParam platform using coding sequence of this gene revealed the protein to have GRAVY of -0.446 and predicted to be extracellular (NLS score = -0.47) (**Figure 8**), consistent with its secretory defense function likely operating in the apoplastic space (Nakai and Horton, 1999). The aliphatic index (50.83) implies modest thermal stability, typical of flexible defense peptides that can function in diverse physiological conditions (Ikai, 1980). BBTI4 exhibits a balanced charge profile (35 acidic vs. 31 basic residues), supporting interactions with both positively and negatively charged pathogen enzymes (Rawlings et al., 2018). The structure exhibits good overall quality, with a MolProbity score of 2.39 reflecting well-refined geometry typical of high-resolution models (Chen et al., 2010). However, the Ramachandran Favored value of 87.86%—slightly below the ideal >90% suggests a few residues occupy less favored conformations, which is acceptable for small, flexible peptides (Sobolev et al., 2020).

**Figure 8:**
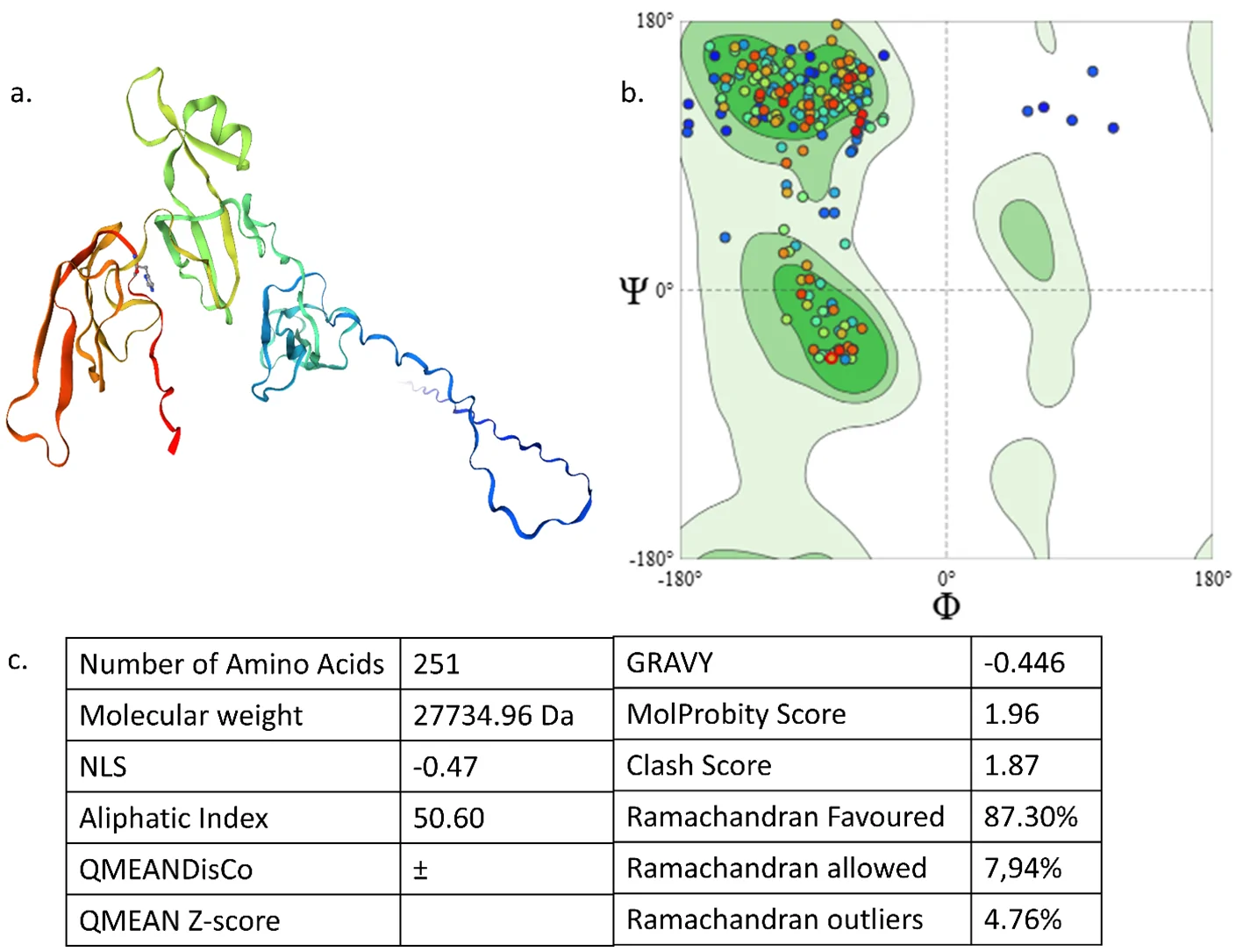
*In silico* structural characterization of the protein encoded by the gene LOC_Os01g03360 (BBPI). **(a)** Predicted 3D structure of the 251-amino-acid peptide (~27.7kDa) generated using SWISS MODEL **(b)** Ramachandran plot illustrating the stereochemical quality of the modeled structure **(c)** Summary of key physicochemical and validation parameters.

### 4.4 Potential synergy and roleplay of the putative candidate genes against sheath blight in rice

The three candidate genes—BBTI4, DEFL14, and AO/AAO—offer complementary mechanisms that could be strategically combined to enhance sheath blight tolerance in rice. BBTI4 directly inhibits pathogen-derived proteases, reducing tissue maceration and slowing infection (Hartl et al., 2011, Chen et al., 2023), while DEFL14 provides extracellular antifungal activity and modulates ROS and defense signaling at infection sites (Carvalho and Gomes, 2009). AO/AAO, by regulating ABA and auxin crosstalk, can fine-tune JA/ET-dependent defenses, shaping the hormonal environment for optimal resistance (Deb et al., 2016; Shi et al., 2021). Together, these genes likely act synergistically, with protease inhibition and antifungal activity directly suppressing pathogen growth, while hormonal modulation ensures that host defense responses are appropriately activated without excessive tissue damage (Li et al., 2021; Yang et al., 2019). However, potential antagonistic interactions may arise from hormone crosstalk; for example, excessive ABA production could counteract JA/ET-mediated pathways induced by BBTI4 and DEFL14 (Deb et al., 2016; Naveenkumar et al., 2022). Therefore, careful regulation of AO/AAO expression, either through tissue-specific promoters or inducible systems, would be critical to maximize synergistic effects while minimizing antagonism (Li et al., 2024). Integrating these genes into multi-gene resistance strategies could provide a robust, multi layered defense framework, combining biochemical, signaling and hormonal barriers against necrotrophic pathogens (Li et al., 2021, 2024).

### 4.5 Potential donors with superior haplotypes for further crop improvement programs

The superior haplotype groups (the haplotypes with lower AUDPC) were filtered to find common entries across the three seasons evaluated in this study. The top five genotypes that were identified as common entries in all three seasons were Rata Boro, Boro, Jagle Boro, Tupa, and Nagri. The performance of these genotypes with respect to their AUDPC levels and their superior haplotype alleles are presented in **Figure 9**. When compared with the tolerant checks, Jasmine-85 and Tetep, the selected superior haplotype donors fared much better against sheath blight infection, particularly in *Wet season* 2018, further confirming the GWAS results and haplotype differences. These lines can further be validated and used as donors for sheath blight tolerance in crop improvement programs.

**Figure 9:**
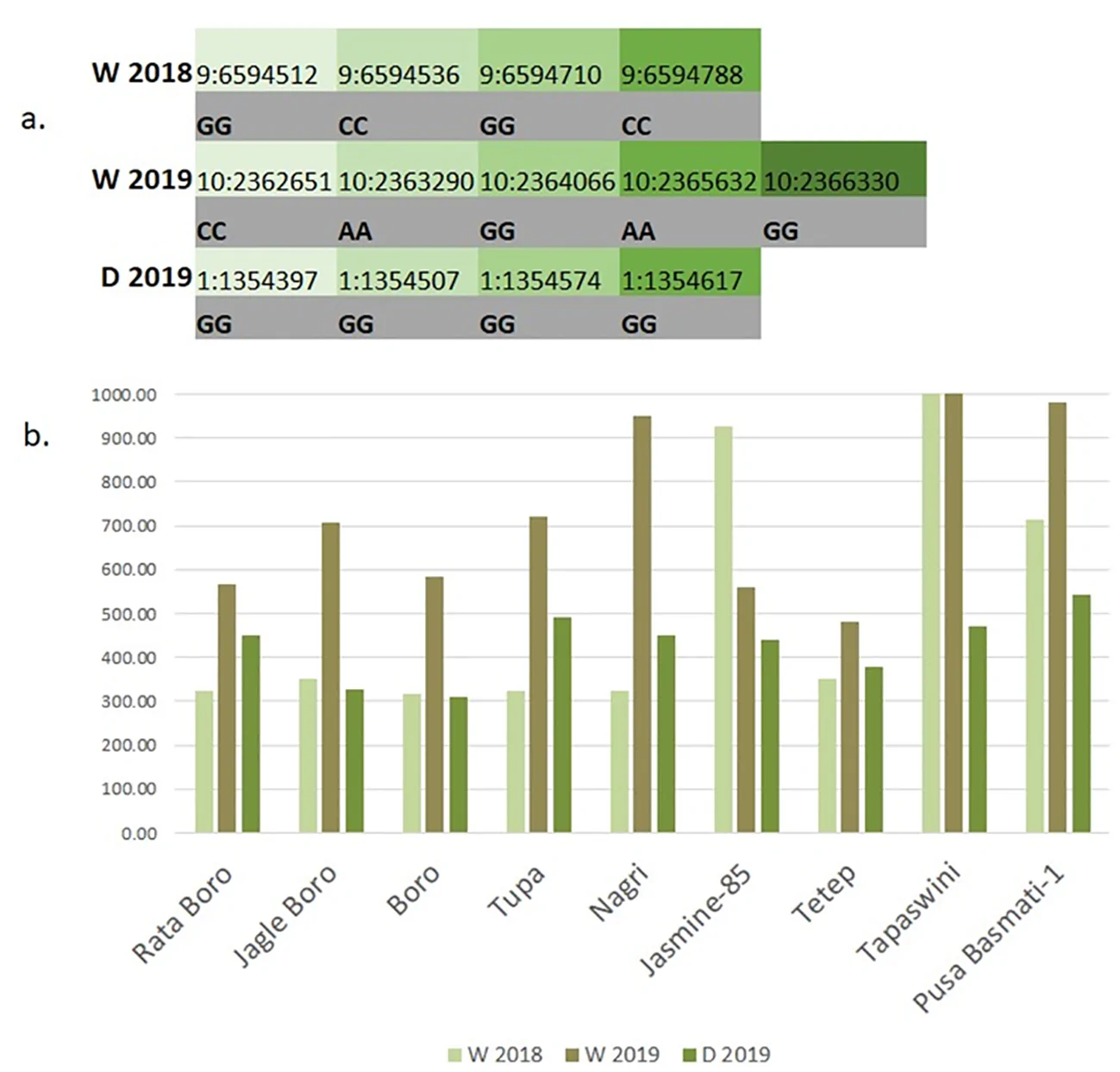
Superior haplotype donors identified across the three experimental seasons. **(a)** Non-synonymous SNP allelic composition of the superior donor genotypes carrying favorable haplotypes across the three candidate genes. **(b)** AUDPC values of the superior donor genotypes compared with susceptible (Tapaswini and Pusa Basmati-1) and tolerant (Tetep and Jasmine 85) reference cultivars across Wet season 2018, Dry season 2018–19, and Wet season 2019. These accessions represent promising donor lines for breeding sheath blight resistance.

## Data Availability

The datasets presented in this study can be found in online repositories. The names of the repository/repositories and accession number(s) can be found in the article/**Supplementary Material**.
